# Epigenetic repression of antiviral genes by SARS-CoV-2 NSP1

**DOI:** 10.1371/journal.pone.0297262

**Published:** 2024-01-26

**Authors:** Dimitrios G. Anastasakis, Daniel Benhalevy, Nicolas Çuburu, Nihal Altan-Bonnet, Markus Hafner

**Affiliations:** 1 RNA Molecular Biology Laboratory, National Institute of Arthritis and Musculoskeletal and Skin Diseases, National Institutes of Health, Bethesda, Maryland, United States of America; 2 Laboratory of Cellular Oncology, National Cancer Institute, National Institutes of Health, Bethesda, Maryland, United States of America; 3 Laboratory of Host-Pathogen Dynamics, National Heart, Lung, and Blood Institute, National Institutes of Health, Bethesda, Maryland, United States of America; National Taiwan University, TAIWAN

## Abstract

The severe acute respiratory syndrome coronavirus 2 (SARS-CoV-2) evades the innate immune machinery through multiple viral proteins, including nonstructural protein 1 (NSP1). While NSP1 is known to suppress translation of host mRNAs, the mechanisms underlying its immune evasion properties remain elusive. By integrating RNA-seq, ribosome footprinting, and ChIP-seq in A549 cells we found that NSP1 predominantly represses transcription of immune-related genes by favoring Histone 3 Lysine 9 dimethylation (H3K9me2). G9a/GLP H3K9 methyltransferase inhibitor UNC0638 restored expression of antiviral genes and restricted SARS-CoV-2 replication. Our multi-omics study unravels an epigenetic mechanism underlying host immune evasion by SARS-CoV-2 NSP1. Elucidating the factors involved in this phenomenon, may have implications for understanding and treating viral infections and other immunomodulatory diseases.

## Introduction

The recent COVID-19 pandemic caused by the severe acute respiratory syndrome coronavirus (SARS-CoV-2) further intensified the study of host-pathogen interactions and the mechanisms through which viruses suppress cellular antiviral responses [1–4]. SARS-CoV-2 evolved elaborate ways to evade the innate immune machinery through the independent action of several of the 29 proteins it encodes. Here, we focused on the multifunctional SARS-CoV-2 nonstructural protein 1 (NSP1), which may contribute to the exceptional pathogenesis of SARS-CoV in humans [5, 6].

Multiple molecular mechanisms for NSP1 function were proposed. Early studies showed that NSP1 selectively suppresses transcription of genes driven by various promotors simian virus 40 (SV40), cytomegalovirus (CMV), interferon (IFN)-b), without affecting actin and rRNA levels [7]. Other reports demonstrate direct suppression of cellular mRNAs by NSP1, either through endonucleolytic cleavage or binding to the 40S scanning ribosomal subunit causing a stall in the mRNA 5’ untranslated regions (UTR) and subsequent mRNA cleavage [4, 8–10]. Recently, NSP1 was shown to globally shut down host mRNA translation [4, 11–13] and compete for binding on the 40S ribosomal subunit [14], further supported by the previous observation that the Transmissible Gastroenteritis Coronavirus (TGEV) NSP1 homolog is directly responsible for host translation shutdown [15].

Of note, SARS-CoV NSP1 selectively suppresses host innate immune functions, including type I IFN expression, and in combination with other viral proteins it inhibits both IFN-β induction and signaling [4, 16]. Compared with SARS-CoV-2 NSP1, SARS-CoV, or MERS-CoV NSP1 homologues have a significantly weaker inhibitory activity towards IFN-α signaling. Given its potent immunomodulatory functions, SARS-CoV2 NSP1 may serve as a promising target for therapeutic intervention.

Here, we combined several high-throughput approaches to probe NSP1 function. Our results suggest that NSP1 is a multifunctional protein that, in addition to affecting the host translational machinery, promotes innate antiviral immune evasion by epigenetic reprogramming. We show that transfection of NSP1 when compared to either transfection of GFP, mutant NSP1 expressing plasmids or empty vectors in A549 cells favored Histone 3 Lysine 9 dimethylation (H3K9me2). Consequently, epigenetic modulation by NSP1 resulted in repression of immune-related genes. Furthermore, treatment with the specific H3K9 methyltransferase inhibitor UNC0638 attenuated the repression of antiviral genes by NSP1. In addition, we show that UNC0638 treatment restored antiviral gene expression and blocked viral replication during SARS-CoV-2 infection of human lung epithelial cells, highlighting the therapeutic potential of epigenetic modulation.

## Materials and methods

### Cell culture

A549 cells (ATCC CCL-185) were cultured in DMEM medium (Gibco) supplemented with 10% (v/v) fetal bovine serum (FBS), 100 μg/ml zeocin, and 10 μg/ml blasticidin (Gibco). pEXP(FLAG/HA-NSP-WT) and pEXP(FLAG/HA-NSP K164A/H165A), (Addgene IDs 188781, and 188782, respectively) were generated using the Gateway system (Invitrogen) as described before [17]. For transient transfection plasmids were transfected into cells using Lipofectamine 3000 (Invitrogen) according to manufacturer’s instructions, expression quantification by immunofluorescence was performed as previously described [18]. Poly(I:C) was transfected with the plasmid at 1/10 of the plasmid (500 ng plasmid per 300,000 cells). RNA from transfected cells was isolated using the Direct-zol RNA Miniprep Kit (Zymo Research, Cat# R2050) according to the manufacturer’s instructions.

### SARS-CoV2 infection

A549 cells stably expressing Angiotensin Converting Enzyme 2 (ACE2) were plated at 50,000 cells/well in a 24 well culture dish [19]. Twenty-four hours later either UNC0638 (1uM final concentration) or DMSO was added to each well. Cells were placed in 37°C, 5% CO_2_ incubator for 1 hour. Subsequently, either 1uL of SARS-CoV2-D614G (4.7e5 TCID50/mL) or 1uL of media was added to each well. Cells were placed back in 37°C, 5% CO_2_ incubator. After 4 hours, virus and drug were washed off the cells and media were replaced. UNC0638 at 1uM concentration or DMSO was added back to wells and cells placed in 37°C, 5% CO_2_ incubator for another 20 hours. At 24 hours post infection cells were lysed in Trizol Ls and RNA lysates processed for qPCR and RNA Sequencing.

### Cytokine measurements

The LEGENDplex Human Anti-Virus Response Panel (biolegend) was used for quantification of 13 human proteins, including IL-1β, IL-6, IL-8, IL-10, IL-12p70, IFN-α2, IFN-β, IFN-λ1, IFN-λ2/3, IFN-γ, TNF-α, IP-10 and GM-CSF directly from cell culture medium.

### Ribosome footprinting

Ribosome footprinting was performed as previously described [20], and quality control analysis was performed using *ribosomeProfilingQC* [21]: A549 cells were seeded on a 10 cm plate at 60% confluence and after 24 hours transfected with 12.5 μg plasmid as described above for expression of NSP1 or GFP. Twelve hours post tr2ansfection cells were supplemented with 100 μg/ml Cycloheximide (CHX) and immediately placed on ice. Media was aspirated, cells were washed with ice-cold PBS, then overlaid with 400 μl of ribosome footprinting buffer (20 mM Tris pH 7.4, 150 mM NaCl, 4 mM MgCl_2_, 1 mM DTT, 100 μg/ml CHX) supplemented with 1% (v/v) NP40 and 25 U/ml Turbo DNaseI, and collected using a rubber policeman into prechilled 1.5 ml microcentrifuge tubes. Extracts were incubated on ice for 10 min, triturated by passing 10 times through a 26-G needle, and cleared of debris by centrifugation at 4°C and 20,000 g for 10 min. Then, 7.5 μl of RNase I (100 U/μl, 2.5 U/μl final concentration) were added to 300 μl of the recovered cell extracts and incubated for 45 min, at room temperature with gentle mixing. Next, 10 μl of SUPERaseIn (20 U/μl) was added in order to stop RNaseI, and the extracts were ultracentrifuged for 4 hours (TLA 100.3 rotor at 70,000 RPM) through a 900 μl sucrose cushion (footprinting buffer with 20 U/ml SUPERasIN and 1 M sucrose). The ultracentrifugation supernatant was discarded, and the ribosome- and footprint-containing pellets were resuspended in 150 μl of footprinting buffer supplemented with 20 U/ml SUPERasIN. RNA was purified by phenol-chloroform extraction, followed by ethanol precipitation. The precipitated RNA was washed twice with 75% ethanol, air dried, resuspended in 15 μl DEPC-treated water and used for construction of NGS sequencing libraries based on previous procedures [22–26] with minor modification described below.

### Ribo-seq NGS library preparation

Five microliters of RNA comprising ribosomes and mRNA footprints were dephosphorylated using quick CIP (NEB) in a 15 μl reaction volume for 15 minutes at 37°C, followed by isolation with Purlink miRNA isolation columns according to manufacturer instruction, with a 1:1 ratio of binding buffer and Ethanol, and concentration to 13 μl volume with the Oligo Clean & Concentrator kit (Zymo Research). Then the dephosphorylated RNA was subjected to 3’ adapter ligation as described in [26]. Essentially, 10 μl of footprints RNA was mixed with 1 μl of 10 μM 5’-adenylated DNA adapter (5’-rAppNNTAGCGATGGAATTCTCGGGTGCCAAGG-L, index sequence underlined and can be varied), 2 μl of 10x RNA ligase buffer without ATP (NEB) and 6 μl of 50% PEG-8000; denatured at 90°C for 1 min, chilled on ice and added with 1 μl of Rnl2(1–249)K227Q RNA ligase (NEB) and 0.5 μl of SUPERaseIN (Thermo Fisher Scientific), and incubated overnight at 4°C.

Ligated RNA was purified using oligo clean and concentrate kit (Zymo) and triple eluted each with 23 μl volume of nuclease-free water. Then 68 μl of the purified ligated RNA was added to 8 ul 10x T4 polynucleotide kinase (PNK), 0.8 μl of 100 mM ATP, and 4 μl of 10 U/μl T4 PNK (NEB) and subjected to 5’ phosphorylation for 30 minutes at 37°C. The 5’-phophorilated 3’-ligated RNA was purified using the Oligo Clean & Concentrator kit (Zymo Research) using 160 μl binding buffer mixed with 320 μl Ethanol, eluted with 25 μl nuclease free water, and subjected to 5’ adapter ligation by addition of 1.25 μl of 50 μM 5’ chimeric DNA-RNA adapter (5’aminolinker-GTTCAGAGTTCTACAGTCCGACGATCrNrNrNrN), 5 μl od 10x RNA ligase buffer with ATP (Thermo) and 15 μl of 50% PEG-8000, denaturation at 90°C for 1 minute followed by immediate cooling on ice, addition of 5 μl of T4 Rnl1 RNA ligase (Thermo Fisher Scientific) and 1 μl of SUPERaseIN (Thermo Fisher Scientific), and incubation at 37°C for 1 hour.

The 3’- 5’- ligated RNA was purified using oligo clean and concentrate kit (Zymo) using 100 μl binding buffer mixed with 200 μl Ethanol, and eluted with 35 μl nuclease-free water, and split into three 0.2 ml tubes of 11 μl each for reverse transcription: Each tube was added with 1 μl of 50 μM RT primer (GCCTTGGCACCCGAGAATTCCA) and 1 μl of 10 mM (each) dNTPs mix, heated to 65°C for 5 minutes and chilled on ice for 1 minute, then added with a mix containing 4 μl of 5X SSIV reaction buffer (Thermo Fisher Scientific), 1 μl of 100 mM DTT, 1 μl of SUPERaseIN, and 1 μl of SuperScript^®^ IV Reverse Transcriptase (200 U/μl, Thermo Fisher Scientific), and incubated at 56°C for 30 minutes, followed by inactivation at 80°C for 10 minutes. The three cDNA-containing reactions were pooled and added with 120 μl nuclease-free water.

The cDNA (120 μl of it) was amplified as described in [24] by addition of 30 μl of 10 mM (each) dNTPs, 36 μl of 50 mM MgCl_2_, 120 μl of 10x PCR reaction buffer, 6 μl of 100 μM 5’-short PCR primer (CTTCAGAGTTCTACAGTCCGACGA), 6 μl of 100 μM RT primer (GCCTTGGCACCCGAGAATTCCA), 4.8 μl of Taq DNA polymerase, and 877.2 μl of nuclease-free water. The reaction was thermo-cycled for a total of five cycles (94°C, 30 s; 60°C, 30 s; 72°C, 15 sec) with a preliminary 2-minute hot start at 94°C and followed by 1 minute at 72°C. Then the PCR product was purified using the DNA Clean & Concentrator™-5 kit (Zymo), eluted with 32 μl of nuclease free water, and size selected (74–88 bp) using 3% agarose Pippin Prep (Sage Science).

A 10 μl sample of the size selected short PCR product was used to assemble a pilot PCR reaction for calibration of the required number of cycles for further amplification. The reaction mix added included 2.5 μl of 10 mM (each) dNTPs mix, 3 μl of 50 mM MgCl_2_, 10 μl of 10x PCR reaction buffer and 0.5 μl of 100 μM 5’-long PCR primer:

(AATGATACGGCGACCACCGAGATCTACACGTTCAGAGTTCTACAGTCCGA),

0.5 μl of 100 μM 3’ indexed primer:

(CAAGCAGAAGACGGCATACGAGATCGTGATGTG ACTGGAGTTCCTTGGCACCCGAGAATTCCA, index is underlined), 0.2 μl of Taq DNA polymerase and 72 μl of nuclease-free water, and the reaction was split to 8 0.2 ml tubes and cycled 6/8/10/12/14/16/18/20 times (94°C, 30 s; 60°C, 30 s; 72°C, 15 sec) with a preliminary 2 minute hot start at 94°C and followed by 1 minute at 72°C. The products were loaded onto a 2.5% agarose gel, size separated by electrophoresis and imaged to select the minimal number of cycles enabling visual representation of the required product. Then 60 μl of the size selected short PCR product were used to assemble an identical reaction mix at larger volume thermo-cycled for the optimal number of times. To the 60 μl of size selected product– 15 μl of 10 mM (each) dNTPs mix, 18 μl of 50 mM MgCl_2_, 60 μl of 10x PCR reaction buffer, 3 μl 0f 100 μM 5’-long PCR primer:

(AATGATACGGCGACCACCGAGATCTACACGTTCAGAGTTCTACAGTCCGA),

3 μl of 100 μM 3’ indexed primer:

(CAAGCAGAAGACGGCATACGAGATCGTGATGTGACTGGAGTTCCTTGGCA CCCGAGAATTCCA, variable index is underlined), 2.4 μl of Taq DNA polymerase and 438 μl of nuclease free water were added and the reaction was thermo-cycled, followed by DNA Clean & Concentrator™-5 kit purification (Zymo Research) purification and elution with 80 μl of nuclease free water. A 1 μl sample of pure libraries was analyzed for size distribution and concentration using TapeStation (Agilent), and if a secondary product of empty library was apparent the libraries were subjected to additional size selection by addition of 84 μl of AMpure XP beads (Beckman Coulter), followed by elution into 20 μl of nuclease free water. Libraries were sequenced on a Illumina HiSeq X Ten. Bcl files were converted to fastq files using bcl2fastq. Adapters were trimmed using cutadapt v 2.4. and reads were mapped to the known transcriptome using tophat2 [27].

### RNA sequencing and qPCR

100 ng total RNA was Ribosomal RNA depleted using the NEBNext® rRNA Depletion Kit and cDNA libraries were prepared using the NEBNext® Ultra™ Directional RNA Library Prep Kit for Illumina® (NEB) according to manufacturer’s instructions. cDNA libraries were sequenced on the Illumina HiSeq 3000, or NovaSeq 6000 platform. Reads were aligned to human genome version hg38 using STAR (star/2.7.2b) [28]. Cufflinks was used for differential expression [27]. cDNA for Real-Time PCR was synthesized using the Real-Time PCR High-Capacity cDNA Reverse Transcription Kit (Applied Biosystems 4368814). Real-Time PCR was performed using the Fast SYBR Green Master Mix (Applied Biosystems 4385610). The following primers were used. IFNB1F: AGTAGGCGACACTGTTCGTG, IFNB1R2: AGCCTCCCATTCAATTGCCA, IFNL1F: GAGGCCCCCAAAAAGGAGTC, IFNL1R5: AGGTTCCCATCGGCCACATA, IFNL2F: TCACGCGAGACCTGAATTGT, IFNL2R: TCTCAGGTTGCATGACTGGT, GAPDHF: CCATGGGGAAGGTGAAGGTC, GAPDHR1: TGATGACCCTTTTGGCTCCC, ACTBF: GTTGTCGACGACGAGCG, ACTBR: GCACAGAGCCTCGCCTT, NSP1F: ATGGAGAGCCTTGTCCCTGG and NSP1R: CCCTCCGTTAAGCTCACGC (NSP1 primers were also used for estimating SARS-CoV2 RNA levels after infection).

### Chromatin immunoprecipitation and sequencing

Two 15 cm plates of transfected A549 were crosslinked by adding paraformaldehyde at a final concentration of 0.75% (Alfa Aesar 43368) for 10 min. Crosslinking was quenched with 125 mM glycine (final) for 5 min. Cells were washed and collected with PBS and lysed with 0.5 ml of lysis buffer (50 mM HEPES-KOH pH7.5, 140 mM NaCl, 1 mM EDTA pH 8.0, 1% Triton X-100, 0.1% Sodium Deoxycholate, 0.1% SDS and protease inhibitor cocktail [cOmplete, Roche]). After sonication using a bath sonicator (Bioruptor, diagenode) for 14 min (medium amplitude, 30 s on, 30 s off) at 4°C. 0.25 ml of lysate was diluted in RIPA buffer (50 mM Tris-HCl pH 8, 150 mM NaCl, 2 mM EDTA pH 8, 1% NP-40, 0.5% Sodium Deoxycholate, 0.1% SDS, protease inhibitor cocktail) to a volume of 2 ml and IP was performed using 5 μg of anti-H3K9me2 (Abcam, ab32521) or anti-PolII CTD repeat YSPTSPS (phospho S2) (Abcam, ab5095) for 1 hour at 4°C. 30 μl of Protein G Dynabeads were equilibrated in the same buffer and added to the samples for 16 hours at 4°C under rotation. Beads were washed once with the following buffers. Low Salt Wash Buffer (0.1% SDS, 1% Triton X-100, 2 mM EDTA, 20 mM Tris-HCl pH 8.0, 150 mM NaCl), High Salt Wash Buffer (0.1% SDS, 1% Triton X-100, 2 mM EDTA, 20 mM Tris-HCl pH 8.0, 500 mM NaCl), LiCl Wash Buffer (0.25 M LiCl, 1% NP-40, 1% Sodium Deoxycholate, 1 mM EDTA, 10 mM Tris-HCl pH 8.0). Elution was performed with 120 μl elution buffer at 30°C for 15 min with elution buffer (1% SDS, 100mM NaHCO_3_). Next, 4.8 μL of 5 M NaCl and 2 μL RNase A/T1 (Thermo EN0551) were added to the eluent and incubated while shaking at 65°C overnight. 2 μL proteinase K (20 mg/mL) and incubate while shaking at 60°C for 1 h. Phenol/chloroform extraction was performed and the sample was cleaned and concentrated using the DNA Clean & Concentrator-5 kit Zymo Research, D013). DNA was prepared for sequencing using the NEBNext Ultra™ II DNA Library Prep Kit for Illumina. Reads were aligned to human genome version hg38 using STAR (star/2.7.2b) [28] and coverage was calculated using bedtools [29].

### Mass spectrometry

Silencing of PRRC2B was confirmed by protein mass spectrometry following the PEPPI-MS protocol [30]. separating lysed cells by SDS/PAGE and used and extracting the proteins from a relatively broad section of the lane expected to include PRRC2B. Using high sensitivity data dependent analysis LC/MS/MS mass spectrometry we analyzed the samples and identified PRRPC2A, PRRPC2B and PRRPC2C in the controlled silenced samples but only PRRPC2A and PRRPC2C in the silenced. Although this was consistent with our expectations only two peptides were detected for PRRPC2B in the single sample and the signals were weak. Due to the sampling problem in data-dependent analysis we were concerned that under sampling might have led to these peptides being missed in the silenced/deleted sample, so we examined the raw MS1 data. We were able to align the two samples using the elution times of shared signals and so examine the spectral features present in the silenced sample and were able to confirm that, even with spectral summing, there was no recognizable isotope cluster for the peptides detected for PRRPC2B in the silenced sample while prominent clusters were present in the control.

### Immunofluorescence

Cells grown in 24 well plates containing Poly-L-Lysin coated coverslips we fixed in 4% paraformaldehyde. After permeabilization using 0.15% Triton X-100 cells were incubated overnight at 4°C with the Anti-PRRC2B Antibody (1:250 dilution, Atlas Antibodies, HPA064301) followed by incubation with secondary antibody (1:500 dilution) conjugated with Alexa Fluor 488 (Thermo Fisher Scientific, Cat# A-11034) and Hoechst 33342 (Invitrogen) labeling. All samples were imaged by confocal microscopy on a Leica SP5 NLO Confocal Microscope (40x oil objective) at room temperature using the same settings.

## Results

**Fig 1:**
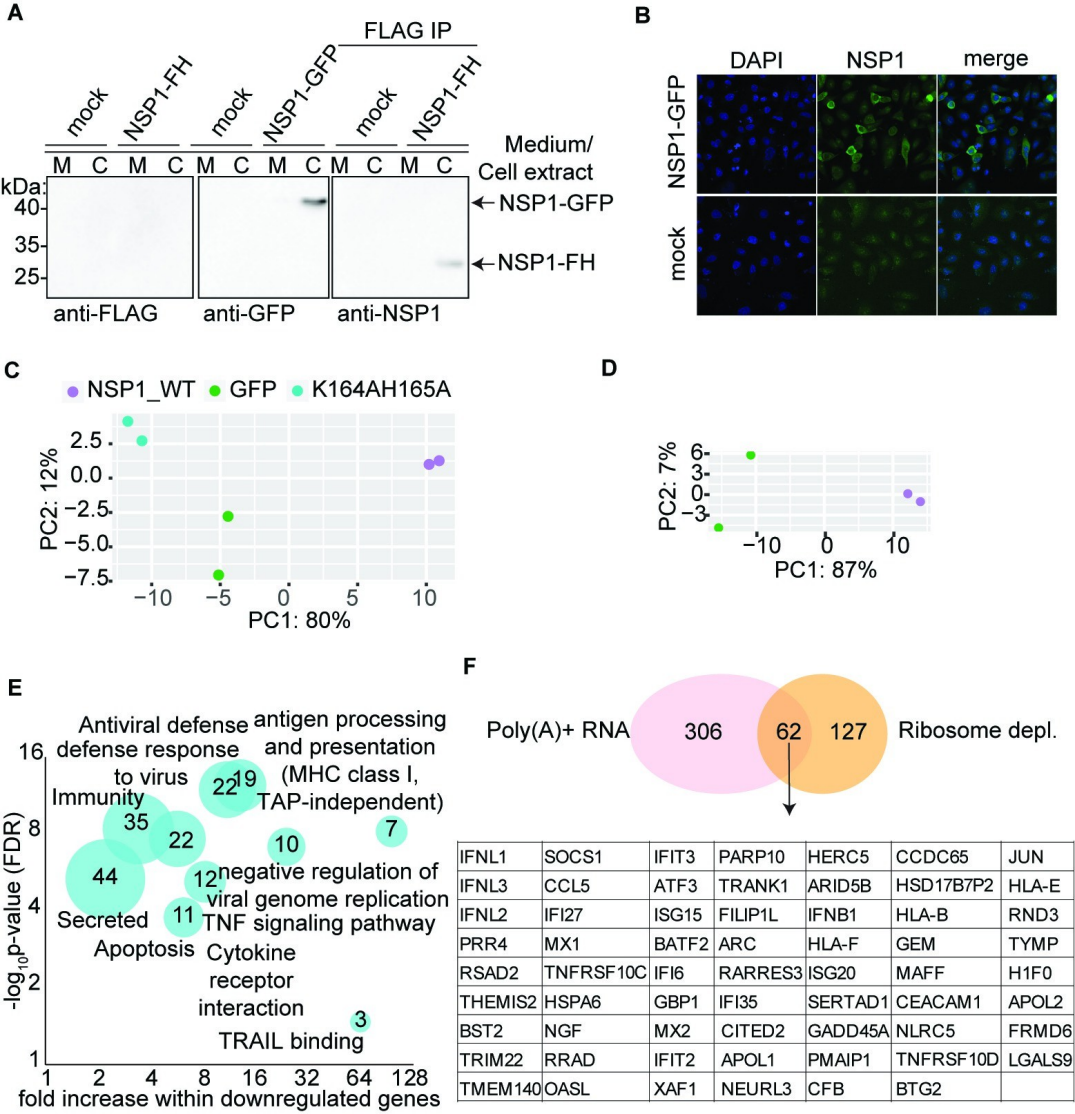
NSP1 downregulates steady-state levels of mRNAs encoded by immune-related genes. **A**.) A549 cells and their growth medium were collected 16 hours after mock, NSP1-GFP or FH-NSP1 transfection. Growth media and cell extracts were analyzed by western blot, and samples from FH-NSP1 cells were also used for immunoprecipitation with anti-FLAG antibodies prior to Western blot analysis using an anti-NSP1 antibody. **B**.) Confocal fluorescence microscopy of A549 cells transfected with NSP1-GFP or mock (same transfection and processing in the absence of DNA). **C,D**) Principal Component Analysis (PCA) of mRNA profiles after transient transfection of NSP1 and control GFP or the K164A/H165A NSP1 mutant, obtained by RNA-seq following rRNA depletion (**C**) or poly-A selection (**D**). **E**.) Gene ontology analysis of transcripts downregulated (≥2-fold) upon expression of NSP1. Size of bubble reflects the number of downregulated genes of the respective categories. **F.)** Venn diagram of genes downregulated by ≥ 2-fold upon NSP1 expression compared to expression of the GFP control, using either rRNA depletion or poly-A selection for mRNA enrichment. Genes shared by the two groups are listed below.

Previous studies indicated that NSP1 globally suppresses host cell mRNA translation by stoichiometrically forming complexes with the mammalian translation machinery [2, 4, 7, 8, 10–12, 14, 31]. Nevertheless, we and others observed that transgenic expression of some NSP1 constructs resulted in cellular toxicity already at low expression levels [31–33], far from matching ribosome stoichiometry. It proved impossible to generate HEK293 and A549 stable cell lines expressing untagged NSP1 or NSP1 fused to a short, unfolded FLAG-HA tag (FH, 1.3 kDa). Even in transiently transfected cells using pFRT-TO-DEST plasmid as backbone the expressed FH-NSP1 transgene remained undetectable by standard Western blotting (Fig 1A). We were only able to detect NSP1 N-terminally fused to the large, globular GFP tag (~17 kDa), in the cytoplasm of A549 cells (consistent with the NSP1-ribosome interaction, Fig 1A and 1B). To confirm that our expression construct produced some levels of FH-NSP1, we therefore concentrated FH-NSP1 by FLAG immunoprecipitation (Fig 1A). We used the same approach to concentrate any FH-NSP1 from the growth media, which ruled out the possibility NSP1 is not detected in cell extracts due to secretion (Fig 1A).

The striking difference of expression levels of untagged or FH-tagged NSP1 compared to NSP1 tagged with a large globular tag leads us to speculate that GFP-tagged NSP1 is either fully or partially non-functional, as often observed for proteins fused to large tags. Considering all the above, we restricted our following cell-based assays to a limited time window of 16–24 hours after transient transfection. Taken together, we find that NSP1 function can be strongly impacted by the choice of affinity tag in cell-based assays. The findings we described in the following suggest a novel role for NSP1 function, manifested at low protein levels that resemble NSP1 levels shortly after SARS-CoV2 cell entry when the protein’s copy number is still far from ribosomal stoichiometry.

To gain insights into the consequences of NSP1 expression on the host cell, we used RNA sequencing (RNA-seq) and compared mRNA profiles of A549 cells expressing FH-NSP1 with cells expressing either a GFP control or a previously reported NSP1 mutant (FH-NSP1 K164A/H165A) [16]. Principal Component Analysis (PCA) of mRNA expression profiles clearly differentiates the transcriptome of NSP1-expressing cells from that of cells expressing GFP or the NSP1 mutant, regardless of whether rRNA depleted RNA (Fig 1C) or poly-A selected RNA (Fig 1D) was used as template for RNA-seq. Gene ontology analysis of differentially expressed mRNAs (≥2-fold relative to GFP expression, after rRNA depletion) showed that NSP1 reduced the steady-state mRNA levels of genes implicated in antiviral defense, antigen processing and presentation (MHC-I, TAP independent), cytokine receptor interaction, and apoptosis (Fig 1E). Sixty-two genes, almost exclusively related to innate immunity were downregulated by more than 2-fold in both poly-A selected, as well as rRNA depleted RNA-seq experiments (Fig 1F). To rule out any bias caused by tagging of NSP we also performed RNA-seq after transfection of untagged NSP1 and observed downregulation of the same 62 genes when compared to either transfection of the nonfunctional untagged NSP1 K164A/H165A or GFP (S1A Fig). These results clearly link a limited amount of NSP1 with a specific suppression of host antiviral response in uninfected cells suggesting a role for NSP1 in reducing antiviral surveillance early during infection (Fig 1E and 1F).

**Fig 2:**
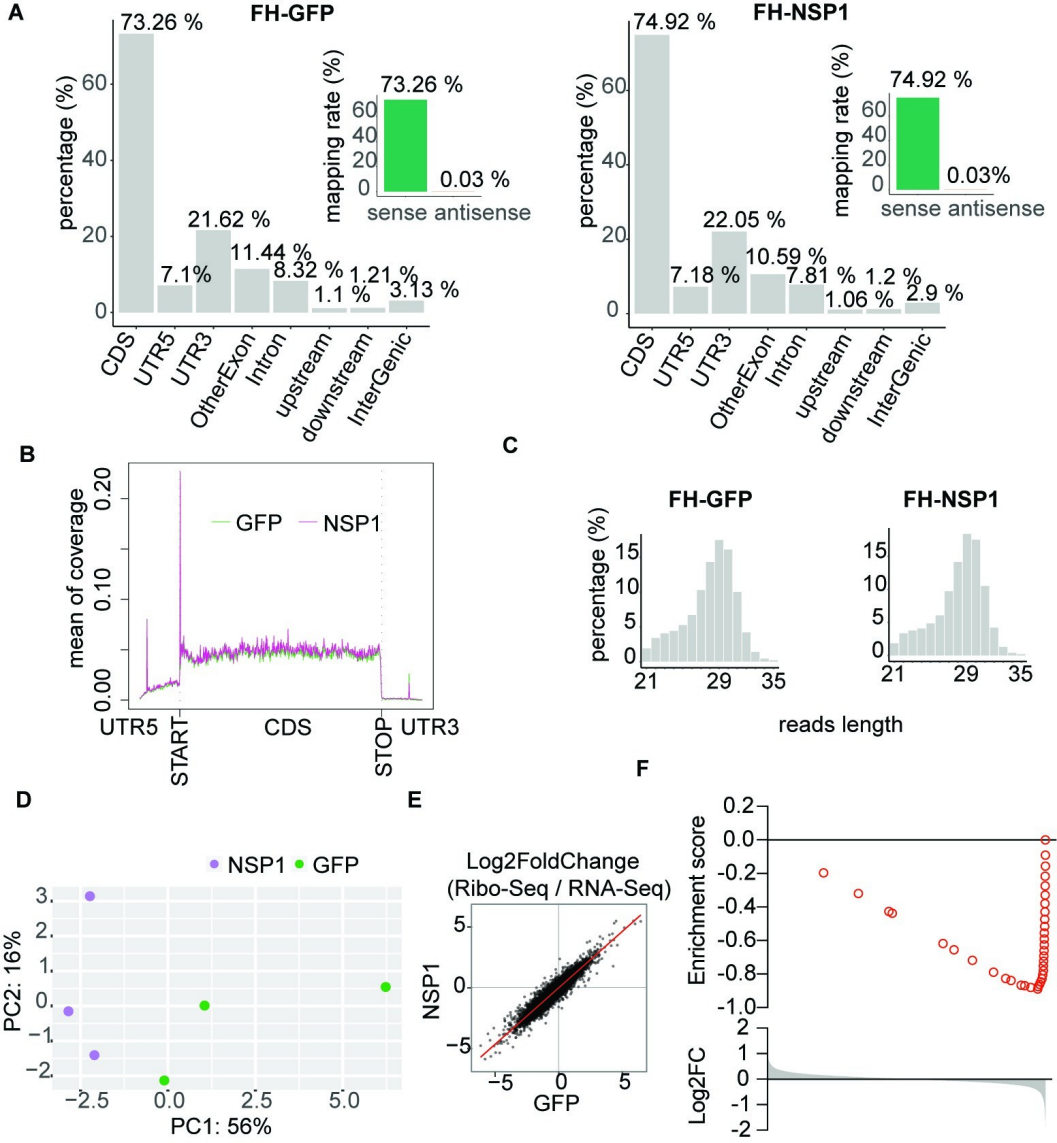
Ribosome footprints follow steady state mRNA abundance. **A**.) Ribo-seq Ribosome-Protected Fragments (RPFs) are as expected aligned predominantly to protein-coding sequences in a sense-strand specific manner. **B**.) Metagene plot showing distribution of ribosome-protected mRNA footprints (RPF) relative to mRNA functional elements. **C**.) RPFs length distribution centers on 29 nucleotides (nt). **D**.) PCA of indicated Ribo-seq experiments. **E**.) Comparison of ribosome density (RPFs versus RPKM) after transient transfection of NSP1 and GFP. Ribo-seq experiments were performed in biological triplicates. **F**.) Gene set enrichment analysis (GSEA) showing that NSP1-mediated changes in RPFs correlate to changes in mRNA abundance.

Considering that NSP1 was previously implicated in regulation of translation, we next assessed the effect of NSP1 expression on translational activity using ribosome footprinting (Ribo-seq) [20, 34] in A549 cells expressing FH-NSP1 or the GFP control. As expected for Ribo-seq experiments, typical Ribosome-Protected Fragments (RPFs) were ~29 nt long, mapped predominantly to mRNA coding sequences (CDS) Fig 2A–2C), and showed the three-nucleotide periodicity characteristic of translating ribosomes (S1B Fig) PCA indicated differential clustering of ribosome-protected mRNA footprints in the presence of NSP1 relative to control expression of GFP (Fig 2D). We noticed that the same mRNAs that were downregulated at the abundance level also showed decreased translation after NSP1 expression and we needed to test whether there was any direct effect of NSP1 on translation, beyond the already significant changes in mRNA abundance level. Therefore, we next approximated translation efficiency per mRNA by normalizing the number of ribosome-protected footprints (RPF) on a given mRNA with its expression level determined by RNA-seq (Fig 2E). We found no change in the translational efficiency of specific genes after NSP1 expression, indicating that—at least 24 hours post transfection—NSP1 has a specific effect on the mRNA abundance of antiviral genes, likely either at the level of transcription or turnover (Fig 2E and 2F).

**Fig 3:**
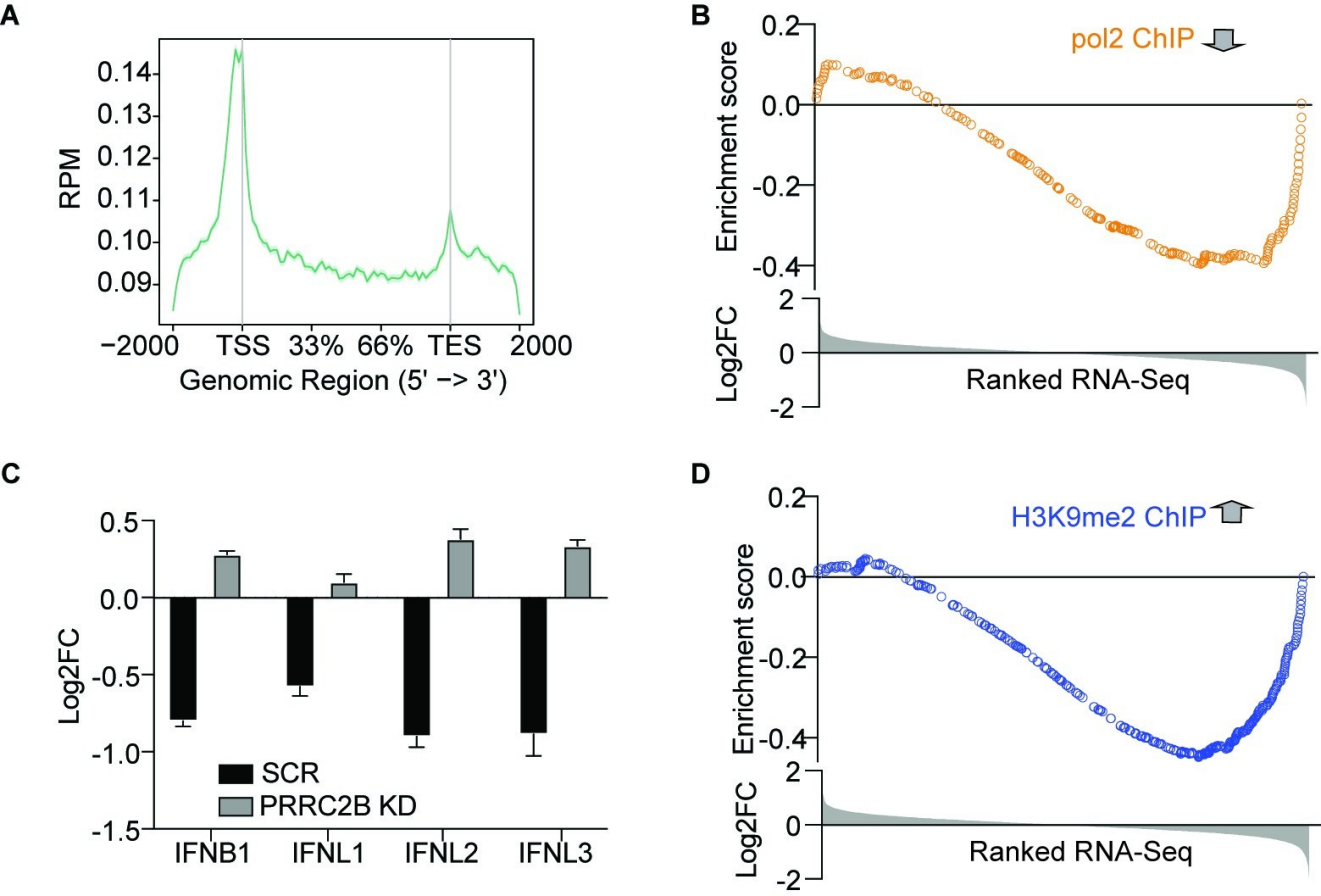
NSP1 affects host-cell transcription of immune-related genes via H3K9me2. **A**.) Meta-analysis of the distribution of PolII associated chromatin (obtained by PolII ChIP-seq) relative to genomic coordinates of protein-coding genes (TSS, Transcription Start Site; TES, Transcription End Site). **B**.) GSEA showing that transcriptionally downregulated genes (PolII ChIP-seq, orange dots) show decreased transcript levels by RNA-seq after NSP1 expression. **C**.) Bar graph of log2Fold change values in RNA-Seq data of WT versus NSP1 K164A/H165A transfected A459 cells after scramble or PRRC2B silencing showing restoration of IFNB1 IFNL1 IFNL2 and IFNL3 expression. **D**.) GSEA showing that genes with increased H3K9me2 mark (blue dots) show decreased transcript levels by RNA-seq after NSP1 expression.

Initially, we favored the hypothesis that NSP1 selectively degrades antiviral gene mRNAs, considering previous reports suggested nucleolytic activity of NSP1. Nevertheless, to rule out a direct effect of NSP1 on transcription we performed RNA Polymerase 2 (PolII) Chromatin Immunoprecipitation and sequencing (ChIP-seq) in cells expressing FH-NSP1 and used the inactive FH-NSP1 K164A/H165A mutant [16] as control. Our PolII ChIP-seq experiments showed the typical chromatin occupancy patterns of actively transcribing PolII, with the expected peaks of signal close to the transcription start sites (TSS) and continuous PolII signal across the gene body (Fig 3A). Next, we integrated PolII occupancy and mRNA abundance changes upon FH-NSP1 expression using Gene Set Enrichment Analysis (GSEA). We found that PolII occupancy changes correlated well with changes in mRNA levels (Fig 3B), strongly suggesting that NSP1 modulates transcription itself, rather than affecting downstream processes, such as RNA stability or translational efficiency.

We hypothesized that with its low expression 24 hours post transfection, NSP1 likely exerted its considerable effect on transcription by epigenetically silencing specific genes. A recent study showed NSP1 interacting with PRRC2B [32], a poorly characterized protein that has been proposed to regulate translation, and also has been found to interact with EHMT2/G9a [35–37]. G9a is a Histone H3 Lysine 9 (H3K9) mono- and di- methyl transferase setting repressive H3K9me and H3K9me2 marks that are known to regulate multiple inflammatory pathways [38–42]. Most importantly, PRRC2B along with the translation initiation factors eIF3a/eIF3G and Ribosomal Proteins RPS10/RPS10 are the only proteins among the strong interactants that lose affinity with the K164A/H165A in both C-terminal and N-terminal NSP1 tagging systems (S2A Fig). This implies that any comparison of the WT vs K164A/H165A may include a PRRC2B-mediatied effect. To assess whether PRRC2B is indeed related to gene regulation by NSP1, we depleted it using siRNAs and confirmed knockdown by immunofluorescence (S2B and S2C Fig) and label-free quantitative mass spectrometry (S1 Table), as PRRC2B could not be detected by immunoblot using commercially available antibodies. PRRC2B knockdown indeed abrogated the NSP1 effect on immune-related mRNAs, compared to control knockdown with scrambled siRNA (Fig 3C), suggesting the possibility that NSP1 may affect histone methylation by G9a through its interaction with PRRC2B. One implication of such indirect effect on G9a is that it dispenses the necessity of NSP1 nuclear entry.

As previously mentioned, H3K9 methylation regulates multiple inflammatory pathways and therefore, we next tested directly whether NSP1 expression influenced H3K9 dimethylation [38, 39]. ChIP-seq with specific anti-H3K9me2 antibodies yielded the expected pattern of chromatin modifications at genic loci (S2D Fig). Genes downregulated by NSP1 show higher levels of the inhibitory H3K9me2 mark after NSP1 transfection (Fig 3D), further supporting that NSP1 might affect host-cell transcription by epigenetic silencing of immune-related loci. While it is unclear whether the effect is mediated fully or in part through PRRC2B, our observations clearly indicate that the effect of NSP1 on gene expression regulation on a global scale is exerted at the level of transcription.

**Fig 4:**
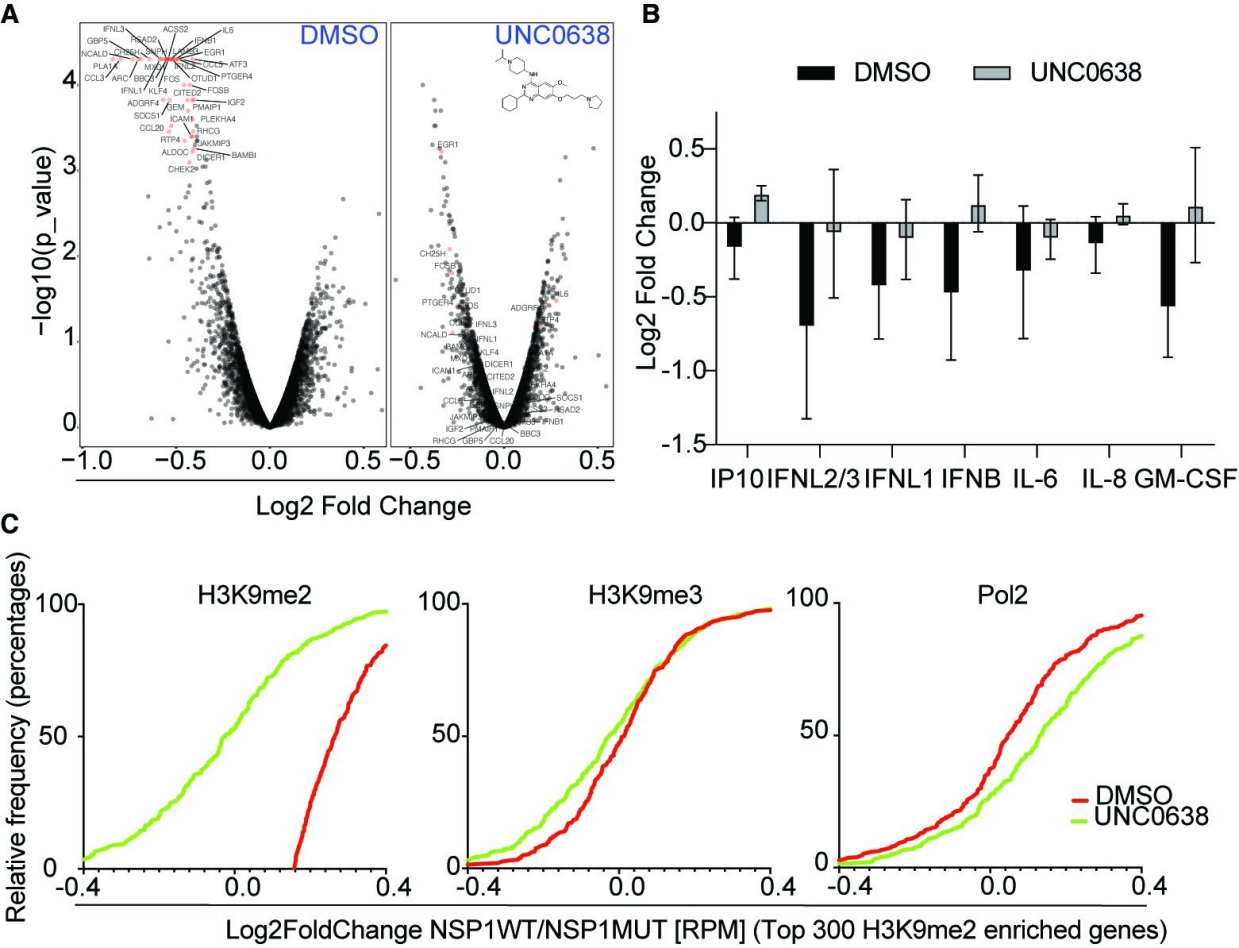
UNC0638 abrogates NSP1-mediated downregulation of immune related. **A**.) Volcano plots showing suppression of type I and III interferon genes by NSP1 (left panel) and attenuation of the suppression following UNC0638 treatment. RNA-seq experiments in each condition are biological triplicates. **B**.) Profiling of secreted cytokines showing restoration of antiviral genes suppressed by NSP1. **C**.) Cumulative distribution frequency plots showing the effect of UNC0638 on H3K9me2 marks (-0.26; *p* value <0.0001), H3K9me3 marks (-0.06; *p* value = 0.075) and PolII occupancy (+0.09; *p* value < 0.001) on the top genes H3K9me2-enriched after NSP1 expression.

Given that NSP1 expression alone, in the absence of viral infection, induced H3K9 methylation and specific downregulation of antiviral mRNAs, we expected that preventing histone methylation of target genes by the methyltransferase G9a would reverse the NSP1-mediated suppression of immune-related genes. To test this, we opted for pharmacological inhibition of G9a by a specific small molecule inhibitor, UNC0638 [43] in the presence of NSP1. RNA-seq analysis showed that UNC0638 completely abrogated NSP1-mediated downregulation of immune related genes (Fig 4A). Furthermore, UNC0638 restored the secretion of antiviral cytokines after stimulation of the interferon pathway by Poly(I:C), which is suppressed by NSP1 (Fig 4B). These findings support a model for histone methylation playing a major role in NSP1-mediated immune evasion. To confirm the effect of UNC0638 on the genes regulated epigenetically by NSP1 we performed CHIP-Seq after treatment. UNC0638 abrogated deposition of H3K9me2 and not of H3K9me3 marks (Fig 4C). The same set of genes showed increased transcription after treatment (Fig 4C).

**Fig 5:**
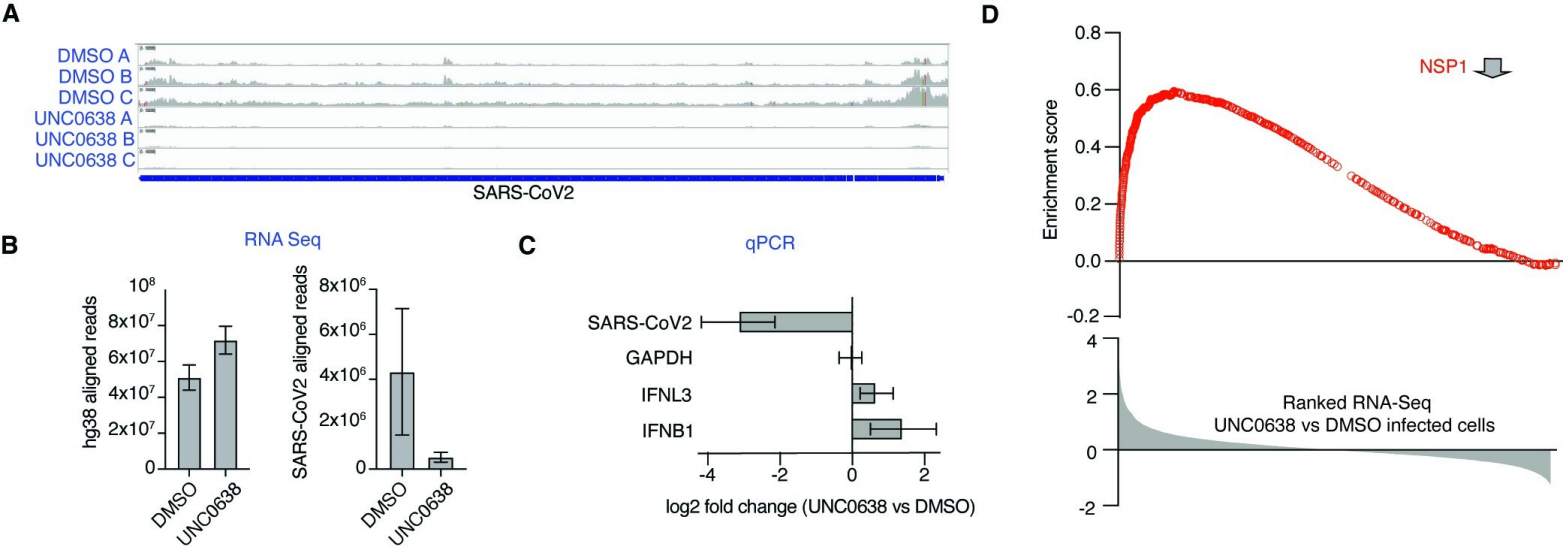
The methyltransferase inhibitor UNC0638 inhibits SARS-CoV2 proliferation in A549 cells. **A**.) IGV plots of SARS-CoV-2 aligned reads from biological replicates of RNA-seq experiments from SARS-CoV-2 infected cells treated with DMSO or UNC0638. **B**.) Absolute numbers of RNA-seq reads aligned to the SARS-CoV-2 (left panel) or the human genome (right panel). **C**.) qPCR showing reduction of viral RNA and induction of IFNB1 and IFNL3 (*n* = 3 for each condition). **D**.) Gene set enrichment analysis (GSEA) showing that transcripts downregulated by FH-NSP1 transfection are induced upon treatment of SARS-CoV-2 infected A549 cells with UNC0638.

With NSP1 promoting the deposition of repressive histone methylation marks on immune-related genes, we thought that inhibition of H3K9 methylation could interfere with the life cycle of SARS-CoV-2. Therefore, we tested UNC0638 in a model of SARS-CoV-2 viral infection of cultured A549-ACE2 cells. We found that treatment with UNC0638 increased expression of innate immune genes and reduced the viral load by ~10 fold, measured by RNA-seq (Fig 5A and 5B) or reverse transcription and quantitative PCR (qPCR) (Fig 5C). Attenuation of immune evasion was confirmed by qPCR showing an increase in IFNB1 and IFNL3 expression (Fig 5C). Finally, GSEA showed that UNC0638 treatment in SARS-CoV-2 infected cells rescued expression of the same transcripts that were downregulated by transient expression of NSP1 in non-infected cells (Fig 5D). In agreement to our results, UNC0638 mediated inhibition of EHMT2 has been shown to induce antiviral response against Foot-and-Mouth disease and Vesicular Stomatitis Virus Infections in bovine cells [44]. Here we provide for the first-time evidence of viral protein-induced host gene suppression via H3K9 dimethylation.

## Discussion

Our results indicate that like many other viral proteins, SARS-CoV-2 NSP1 is multifunctional. In addition to previously reported functions, including its well-documented role in translational repression [3–5, 7–16, 31, 45, 46], SARS-CoV-2 NSP1 may suppress host innate immune genes by epigenetic reprogramming. We propose that early in infection, while still at low copy number, NSP1 induces G9a-mediated H3K9 methylation of specific host gene loci, resulting in downregulated transcription of immune-related genes and reduced antiviral surveillance. Epigenetic silencing of antiviral genes early upon infection may cause the discrepancy between the levels of type I/III interferons and proinflammatory cytokines observed in COVID-19 patients. Compared to influenza patients, induction of both IFN-λ and type I IFNs is both impaired and delayed in patients with COVID-19 while pro-inflammatory cytokines are detected at similar levels [47]. This imbalance is the main reason SARS-CoV-2 can delay antiviral response and persist for a long period of time. Because severe symptoms are caused by high levels of pro-inflammatory cytokines and tissue damage, it is highly unlikely that inhibition of histone methylation can be used for therapy. However, aerosol-delivered H3K9 methyltransferase inhibitors could potentially have a beneficial effect prior or early after viral exposure and act as preventive drugs for frontline health-care workers combating an outbreak. Although UNC0638 has poor pharmacokinetic properties, the related UNC0642 has improved *in vivo* characteristics [48].

## Conclusions

The fact that expression of a single viral protein alone induced a viral infection specific phenotype in the absence of viral infection provides confidence in our conclusions. It is unlikely that with the low copy numbers we have in our expression system, NSP1 is able to stoichiometrically interact and inhibit the ribosome. The observed changes in translational output measured by Ribo-seq and RNA-seq can be completely attributed to changes in mRNA levels. We do not contradict that global translational shutdown occurs at a later point of infection. Nevertheless, we propose that at the global scale immune-related genes are downregulated at the transcriptional level by NSP1. H3K9me2 marks are associated with heterochromatin and are involved in chromatin organization unlike transcription factors that can be directly involved in gene promoter activity [49, 50]. In this study, we show that NSP1 favors H3K9me2 marks either by inducing dimethylation or by preventing dynamic demethylation during antiviral response. Consequently, the expression of genes within epigenetically modulated genomic loci are repressed by NSP1. Antiviral response involves chromatin regulation directly and indirectly by production of IFNs and thus, further studies are needed to shed light on which of the genes are primary targets for epigenetic regulation by NSP1 [51]. In addition, during viral infection a multitude of additional processes alter the cell environment and may reduce the physiological relevance of our hypothesis. Nevertheless, we show that inhibition of H3K9 methyltransferase activity using UNC0638 restores antiviral response and inhibits SARS-CoV-2 replication in A549-ACE2. Similarly, it can induce antiviral response against foot-and-mouth disease and vesicular stomatitis virus [44]. In addition, H3K9 dimethylation safeguards cancer cells against activation of the interferon pathway, altogether highlighting the importance of H3K9me2 in immunomodulatory phenomena [52].

## Supporting information

S1 Fig **A.** Log2FoldChange dot blot A549 cells transfected with plasmids expressing untagged NSP1 show similar repression of antiviral genes when compared to cells transfected with empty vector or GFP as control **B.** Ribo-seq Ribosome Protected Fragments (RPFs) align to open reading frames with the expected triplet periodicity.
(TIF)

S2 Fig **A**. Spectral counts from SARS-CoV-2 host proximity interactome data (covid19interactome.org) **B,C.** Confocal Immunofluorescence microscopy analysis of A549 cells after introduction of a scrambled siRNA or an siRNA targeting PRRC2B expression. Green, anti PRRC2B; blue, DAPI. **D.** Meta-analysis of the distribution of di-methylated Histone 3 Lysin 9 chromatin (H3K9me2), obtained by H3K9me2 ChIP-seq, relative to genomic coordinates of protein-coding genes.
(TIF)

S1 Table (TXT)

S1 Raw images (PDF)

## References

[1] Lee JS, Park S, Jeong HW, Ahn JY, Choi SJ, Lee H, et al. Immunophenotyping of COVID-19 and influenza highlights the role of type I interferons in development of severe COVID-19. Sci Immunol. 2020;5(49). doi: 10.1126/sciimmunol.abd1554 32651212 PMC7402635

[2] Banerjee AK, Blanco MR, Bruce EA, Honson DD, Chen LM, Chow A, et al. SARS-CoV-2 Disrupts Splicing, Translation, and Protein Trafficking to Suppress Host Defenses. Cell. 2020;183(5):1325–39 e21. doi: 10.1016/j.cell.2020.10.004 33080218 PMC7543886

[3] Zhang K, Miorin L, Makio T, Dehghan I, Gao S, Xie Y, et al. Nsp1 protein of SARS-CoV-2 disrupts the mRNA export machinery to inhibit host gene expression. Sci Adv. 2021;7(6). doi: 10.1126/sciadv.abe7386 33547084 PMC7864571

[4] Fisher T, Gluck A, Narayanan K, Kuroda M, Nachshon A, Hsu JC, et al. Parsing the role of NSP1 in SARS-CoV-2 infection. Cell Rep. 2022;39(11):110954. doi: 10.1016/j.celrep.2022.110954 35671758 PMC9133101

[5] Connor RF, Roper RL. Unique SARS-CoV protein nsp1: bioinformatics, biochemistry and potential effects on virulence. Trends Microbiol. 2007;15(2):51–3. doi: 10.1016/j.tim.2006.12.005 17207625 PMC7127589

[6] Skowronski DM, Astell C, Brunham RC, Low DE, Petric M, Roper RL, et al. Severe acute respiratory syndrome (SARS): a year in review. Annu Rev Med. 2005;56:357–81. doi: 10.1146/annurev.med.56.091103.134135 15660517

[7] Kamitani W, Huang C, Narayanan K, Lokugamage KG, Makino S. A two-pronged strategy to suppress host protein synthesis by SARS coronavirus Nsp1 protein. Nat Struct Mol Biol. 2009;16(11):1134–40. doi: 10.1038/nsmb.1680 19838190 PMC2784181

[8] Tanaka T, Kamitani W, DeDiego ML, Enjuanes L, Matsuura Y. Severe acute respiratory syndrome coronavirus nsp1 facilitates efficient propagation in cells through a specific translational shutoff of host mRNA. J Virol. 2012;86(20):11128–37. doi: 10.1128/JVI.01700-12 22855488 PMC3457165

[9] Huang C, Lokugamage KG, Rozovics JM, Narayanan K, Semler BL, Makino S. SARS coronavirus nsp1 protein induces template-dependent endonucleolytic cleavage of mRNAs: viral mRNAs are resistant to nsp1-induced RNA cleavage. PLoS Pathog. 2011;7(12):e1002433. doi: 10.1371/journal.ppat.1002433 22174690 PMC3234236

[10] Schubert K, Karousis ED, Jomaa A, Scaiola A, Echeverria B, Gurzeler LA, et al. SARS-CoV-2 Nsp1 binds the ribosomal mRNA channel to inhibit translation. Nat Struct Mol Biol. 2020;27(10):959–66. doi: 10.1038/s41594-020-0511-8 32908316

[11] Thoms M, Buschauer R, Ameismeier M, Koepke L, Denk T, Hirschenberger M, et al. Structural basis for translational shutdown and immune evasion by the Nsp1 protein of SARS-CoV-2. Science. 2020;369(6508):1249–55. doi: 10.1126/science.abc8665 32680882 PMC7402621

[12] Shi M, Wang L, Fontana P, Vora S, Zhang Y, Fu TM, et al. SARS-CoV-2 Nsp1 suppresses host but not viral translation through a bipartite mechanism. bioRxiv. 2020.

[13] Rao S, Hoskins I, Tonn T, Garcia PD, Ozadam H, Sarinay Cenik E, et al. Genes with 5’ terminal oligopyrimidine tracts preferentially escape global suppression of translation by the SARS-CoV-2 Nsp1 protein. RNA. 2021;27(9):1025–45. doi: 10.1261/rna.078661.120 34127534 PMC8370740

[14] Lapointe CP, Grosely R, Johnson AG, Wang J, Fernández IS, Puglisi JD. Dynamic competition between SARS-CoV-2 NSP1 and mRNA on the human ribosome inhibits translation initiation. Proc Natl Acad Sci U S A. 2021;118(6). doi: 10.1073/pnas.2017715118 33479166 PMC8017934

[15] Shen Z, Wang G, Yang Y, Shi J, Fang L, Li F, et al. A conserved region of nonstructural protein 1 from alphacoronaviruses inhibits host gene expression and is critical for viral virulence. J Biol Chem. 2019;294(37):13606–18. doi: 10.1074/jbc.RA119.009713 31350335 PMC6746460

[16] Narayanan K, Huang C, Lokugamage K, Kamitani W, Ikegami T, Tseng CT, et al. Severe acute respiratory syndrome coronavirus nsp1 suppresses host gene expression, including that of type I interferon, in infected cells. J Virol. 2008;82(9):4471–9. doi: 10.1128/JVI.02472-07 18305050 PMC2293030

[17] Landthaler M, Gaidatzis D, Rothballer A, Chen PY, Soll SJ, Dinic L, et al. Molecular characterization of human Argonaute-containing ribonucleoprotein complexes and their bound target mRNAs. Rna. 2008;14(12):2580–96. doi: 10.1261/rna.1351608 18978028 PMC2590962

[18] Kanfer G, Sarraf SA, Maman Y, Baldwin H, Dominguez-Martin E, Johnson KR, et al. Image-based pooled whole-genome CRISPRi screening for subcellular phenotypes. J Cell Biol. 2021;220(2). doi: 10.1083/jcb.202006180 33464298 PMC7816647

[19] López-Muñoz AD, Kosik I, Holly J, Yewdell JW. Cell surface SARS-CoV-2 nucleocapsid protein modulates innate and adaptive immunity. Science Advances. 2022;8(31):eabp9770. doi: 10.1126/sciadv.abp9770 35921414 PMC9348789

[20] Ingolia NT, Brar GA, Rouskin S, McGeachy AM, Weissman JS. The ribosome profiling strategy for monitoring translation in vivo by deep sequencing of ribosome-protected mRNA fragments. Nature Protocols. 2012;7(8):1534–50. doi: 10.1038/nprot.2012.086 22836135 PMC3535016

[21] Ou J. HM. ribosomeProfilingQC: Ribosome Profiling Quality Control. R package version 1.8.0. 2022.

[22] Benhalevy D, Gupta SK, Danan CH, Ghosal S, Sun HW, Kazemier HG, et al. The Human CCHC-type Zinc Finger Nucleic Acid-Binding Protein Binds G-Rich Elements in Target mRNA Coding Sequences and Promotes Translation. Cell Rep. 2017;18(12):2979–90. doi: 10.1016/j.celrep.2017.02.080 28329689 PMC5393907

[23] Benhalevy D, McFarland HL, Sarshad AA, Hafner M. PAR-CLIP and streamlined small RNA cDNA library preparation protocol for the identification of RNA binding protein target sites. Methods. 2017;118–119:41–9. 27871973 10.1016/j.ymeth.2016.11.009 PMC11145949

[24] Anastasakis DG, Jacob A, Konstantinidou P, Meguro K, Claypool D, Cekan P, et al. A non-radioactive, improved PAR-CLIP and small RNA cDNA library preparation protocol. Nucleic Acids Res. 2021;49(8):e45. doi: 10.1093/nar/gkab011 33503264 PMC8096255

[25] Hafner M, Renwick N, Farazi TA, Mihailović A, Pena JT, Tuschl T. Barcoded cDNA library preparation for small RNA profiling by next-generation sequencing. Methods. 2012;58(2):164–70. doi: 10.1016/j.ymeth.2012.07.030 22885844 PMC3508525

[26] Anastasakis D, Benhalevy D, Hafner M. Proximity-CLIP and Expedited Non-Radioactive Library Preparation of Small RNA Footprints for Next-Generation Sequencing. Curr Protoc Mol Biol. 2020;131(1):e120. doi: 10.1002/cpmb.120 32438484 PMC7316200

[27] Trapnell C, Hendrickson DG, Sauvageau M, Goff L, Rinn JL, Pachter L. Differential analysis of gene regulation at transcript resolution with RNA-seq. Nat Biotechnol. 2013;31(1):46–53. doi: 10.1038/nbt.2450 23222703 PMC3869392

[28] Dobin A, Davis CA, Schlesinger F, Drenkow J, Zaleski C, Jha S, et al. STAR: ultrafast universal RNA-seq aligner. Bioinformatics. 2013;29(1):15–21. doi: 10.1093/bioinformatics/bts635 23104886 PMC3530905

[29] Quinlan AR, Hall IM. BEDTools: a flexible suite of utilities for comparing genomic features. Bioinformatics. 2010;26(6):841–2. doi: 10.1093/bioinformatics/btq033 20110278 PMC2832824

[30] Takemori A, Butcher DS, Harman VM, Brownridge P, Shima K, Higo D, et al. PEPPI-MS: Polyacrylamide-Gel-Based Prefractionation for Analysis of Intact Proteoforms and Protein Complexes by Mass Spectrometry. J Proteome Res. 2020;19(9):3779–91. doi: 10.1021/acs.jproteome.0c00303 32538093 PMC8141340

[31] Yuan S, Peng L, Park JJ, Hu Y, Devarkar SC, Dong MB, et al. Nonstructural Protein 1 of SARS-CoV-2 Is a Potent Pathogenicity Factor Redirecting Host Protein Synthesis Machinery toward Viral RNA. Mol Cell. 2020;80(6):1055–66 e6. doi: 10.1016/j.molcel.2020.10.034 33188728 PMC7833686

[32] Samavarchi-Tehrani P, Abdouni H, Knight JDR, Astori A, Samson R, Lin Z-Y, et al. A SARS-CoV-2 –host proximity interactome. bioRxiv. 2020:2020.09.03.282103.

[33] May DG, Martin-Sancho L, Anschau V, Liu S, Chrisopulos RJ, Scott KL, et al. A BioID-derived proximity interactome for SARS-CoV-2 proteins. bioRxiv. 2021. 10.3390/v14030611 PMC8951556 35337019

[34] Ingolia NT, Ghaemmaghami S, Newman JR, Weissman JS. Genome-wide analysis in vivo of translation with nucleotide resolution using ribosome profiling. Science. 2009;324(5924):218–23. doi: 10.1126/science.1168978 19213877 PMC2746483

[35] Rual J-F, Venkatesan K, Hao T, Hirozane-Kishikawa T, Dricot A, Li N, et al. Towards a proteome-scale map of the human protein–protein interaction network. Nature. 2005;437(7062):1173–8. doi: 10.1038/nature04209 16189514

[36] Ito T, Chiba T, Ozawa R, Yoshida M, Hattori M, Sakaki Y. A comprehensive two-hybrid analysis to explore the yeast protein interactome. Proc Natl Acad Sci U S A. 2001;98(8):4569–74. doi: 10.1073/pnas.061034498 11283351 PMC31875

[37] Jiang F, Hedaya OM, Khor E, Wu J, Auguste M, Yao P. RNA binding protein PRRC2B mediates translation of specific mRNAs and regulates cell cycle progression. Nucleic Acids Res. 2023;51(11):5831–46. doi: 10.1093/nar/gkad322 37125639 PMC10287950

[38] Ren X, Wang R, Yu X, Cai B, Guo F, editors. Regulation of histone H3 lysine 9 methylation in inflammation 2021.

[39] Fang TC, Schaefer U, Mecklenbrauker I, Stienen A, Dewell S, Chen MS, et al. Histone H3 lysine 9 di-methylation as an epigenetic signature of the interferon response. J Exp Med. 2012;209(4):661–9. doi: 10.1084/jem.20112343 22412156 PMC3328357

[40] Jit BP, Qazi S, Arya R, Srivastava A, Gupta N, Sharma A. An immune epigenetic insight to COVID-19 infection. Epigenomics. 2021;13(6):465–80. doi: 10.2217/epi-2020-0349 33685230 PMC7958646

[41] Mourits VP, van Puffelen JH, Novakovic B, Bruno M, Ferreira AV, Arts RJ, et al. Lysine methyltransferase G9a is an important modulator of trained immunity. Clin Transl Immunology. 2021;10(2):e1253. doi: 10.1002/cti2.1253 33708384 PMC7890679

[42] Scheer S, Zaph C. The Lysine Methyltransferase G9a in Immune Cell Differentiation and Function. Front Immunol. 2017;8:429. doi: 10.3389/fimmu.2017.00429 28443098 PMC5387087

[43] Vedadi M, Barsyte-Lovejoy D, Liu F, Rival-Gervier S, Allali-Hassani A, Labrie V, et al. A chemical probe selectively inhibits G9a and GLP methyltransferase activity in cells. Nat Chem Biol. 2011;7(8):566–74. doi: 10.1038/nchembio.599 21743462 PMC3184254

[44] Singh N, Ramĩrez-Carvajal L, de Los Santos T, Golding MC, Long CR. Inhibition of EHMT2 Induces a Robust Antiviral Response Against Foot-and-Mouth Disease and Vesicular Stomatitis Virus Infections in Bovine Cells. J Interferon Cytokine Res. 2016;36(1):37–47. doi: 10.1089/jir.2015.0006 26418342 PMC4722570

[45] Kamitani W, Narayanan K, Huang C, Lokugamage K, Ikegami T, Ito N, et al. Severe acute respiratory syndrome coronavirus nsp1 protein suppresses host gene expression by promoting host mRNA degradation. Proc Natl Acad Sci U S A. 2006;103(34):12885–90. doi: 10.1073/pnas.0603144103 16912115 PMC1568942

[46] Xia H, Cao Z, Xie X, Zhang X, Chen JY, Wang H, et al. Evasion of Type I Interferon by SARS-CoV-2. Cell Rep. 2020;33(1):108234. doi: 10.1016/j.celrep.2020.108234 32979938 PMC7501843

[47] Galani I-E, Rovina N, Lampropoulou V, Triantafyllia V, Manioudaki M, Pavlos E, et al. Untuned antiviral immunity in COVID-19 revealed by temporal type I/III interferon patterns and flu comparison. Nature Immunology. 2021;22(1):32–40. doi: 10.1038/s41590-020-00840-x 33277638

[48] Liu F, Barsyte-Lovejoy D, Li F, Xiong Y, Korboukh V, Huang XP, et al. Discovery of an in vivo chemical probe of the lysine methyltransferases G9a and GLP. J Med Chem. 2013;56(21):8931–42. doi: 10.1021/jm401480r 24102134 PMC3880643

[49] Padeken J, Methot SP, Gasser SM. Establishment of H3K9-methylated heterochromatin and its functions in tissue differentiation and maintenance. Nature Reviews Molecular Cell Biology. 2022;23(9):623–40. doi: 10.1038/s41580-022-00483-w 35562425 PMC9099300

[50] Poleshko A, Smith CL, Nguyen SC, Sivaramakrishnan P, Wong KG, Murray JI, et al. H3K9me2 orchestrates inheritance of spatial positioning of peripheral heterochromatin through mitosis. Elife. 2019;8. doi: 10.7554/eLife.49278 31573510 PMC6795522

[51] Au-Yeung N, Horvath CM. Transcriptional and chromatin regulation in interferon and innate antiviral gene expression. Cytokine Growth Factor Rev. 2018;44:11–7. doi: 10.1016/j.cytogfr.2018.10.003 30509403 PMC6281172

[52] Hansen AM, Ge Y, Schuster MB, Pundhir S, Jakobsen JS, Kalvisa A, et al. H3K9 dimethylation safeguards cancer cells against activation of the interferon pathway. Sci Adv. 2022;8(11):eabf8627. doi: 10.1126/sciadv.abf8627 35302840 PMC8932663

