# Supplementary figures and images for: Epigenetic repression of antiviral genes by SARS-CoV-2 NSP1
Source: PLoS One. 2024 Jan 26;19(1):e0297262. doi: 10.1371/journal.pone.0297262 (PMC10817131; doi:10.1371/journal.pone.0297262)

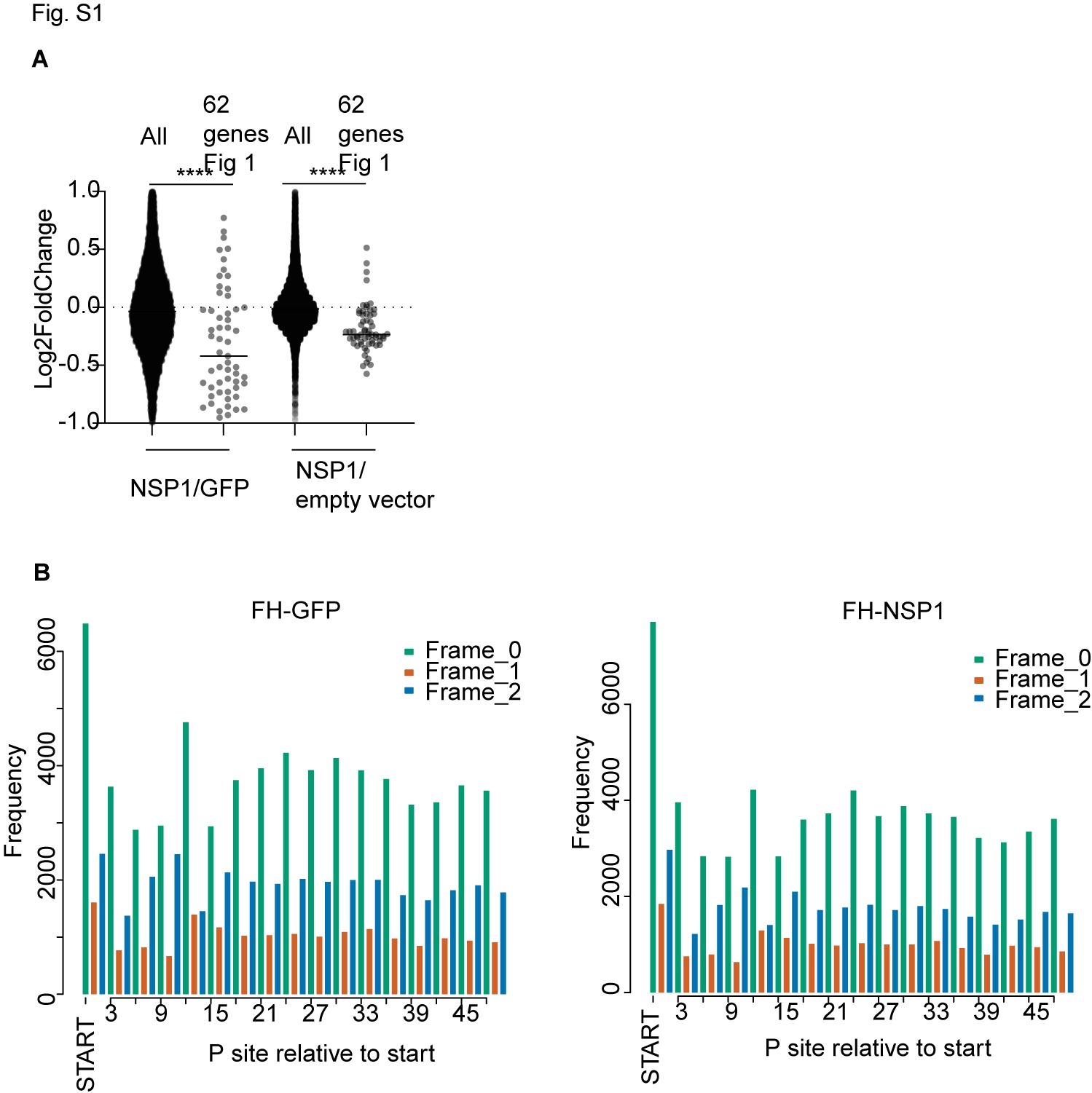

Supplement: S1 Fig — A. Log2FoldChange dot blot A549 cells transfected with plasmids expressing untagged NSP1 show similar repression of antiviral genes when compared to cells transfected with empty vector or GFP as control B. Ribo-seq Ribosome Protected Fragments (RPFs) align to open reading frames with the expected triplet periodicity. (TIF) [file pone.0297262.s001.tif]

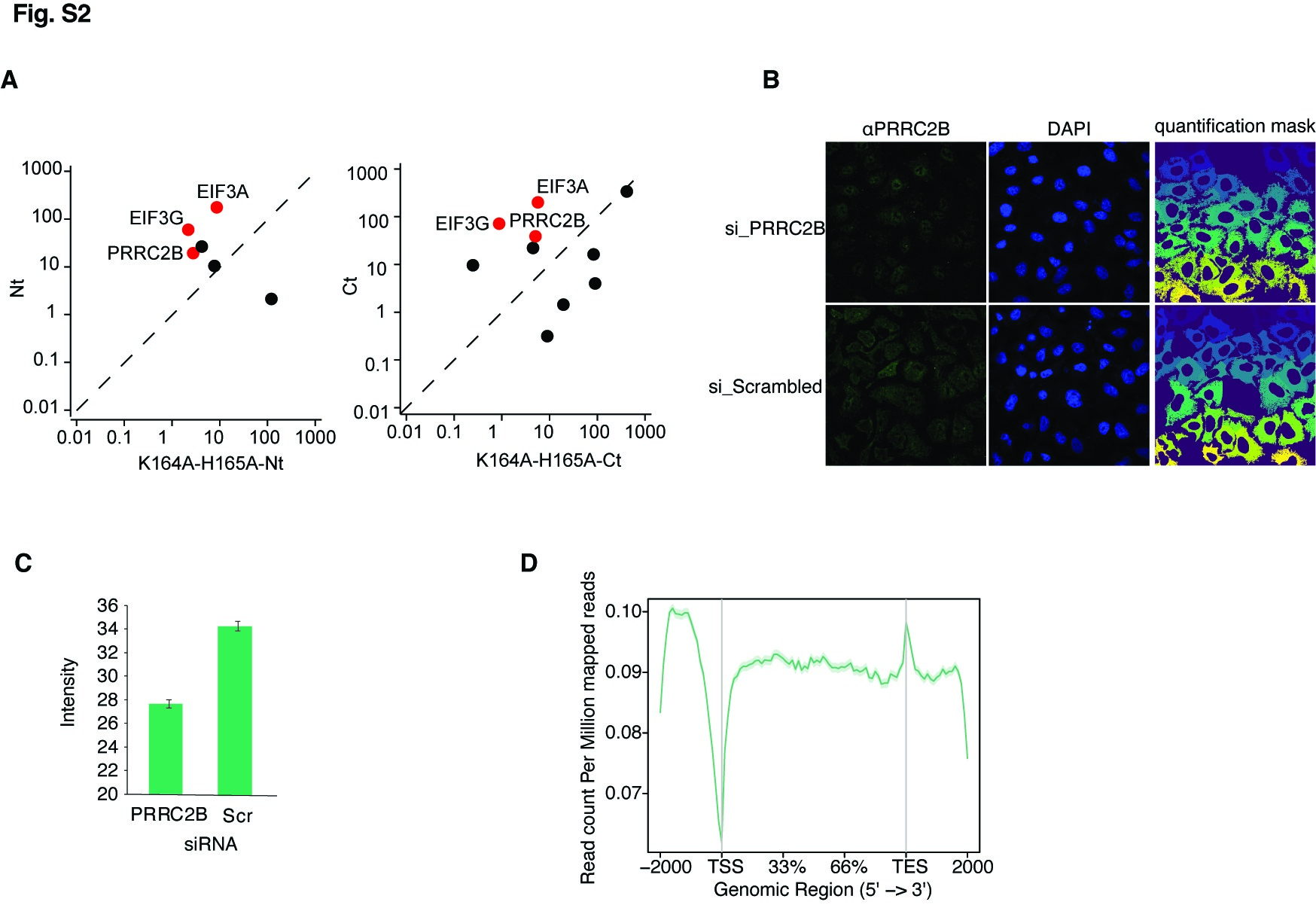

Supplement: S2 Fig — A. Spectral counts from SARS-CoV-2 host proximity interactome data (covid19interactome.org) B,C. Confocal Immunofluorescence microscopy analysis of A549 cells after introduction of a scrambled siRNA or an siRNA targeting PRRC2B expression. Green, anti PRRC2B; blue, DAPI. D. Meta-analysis of the distribution of di-methylated Histone 3 Lysin 9 chromatin (H3K9me2), obtained by H3K9me2 ChIP-seq, relative to genomic coordinates of protein-coding genes. (TIF) [file pone.0297262.s002.tif]

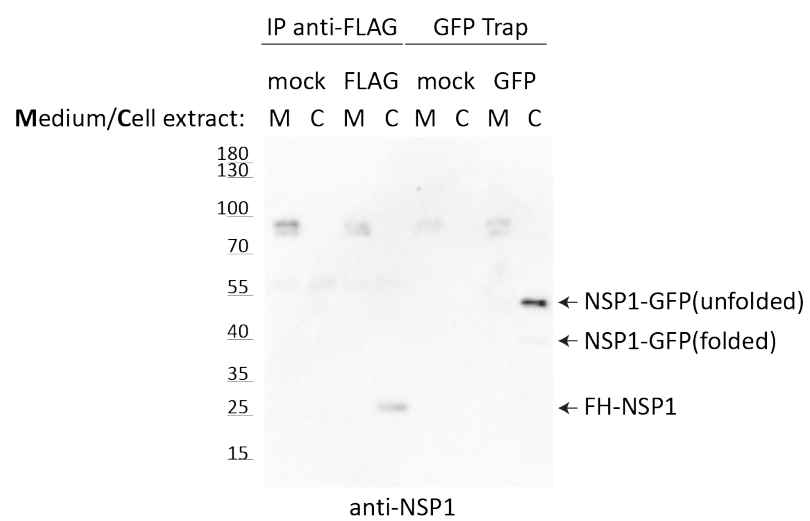

Supplement: S1 Raw images — (PDF) [file pone.0297262.s004.pdf]
